# ResCap: plant resistance gene prediction and probe generation pipeline for resistance gene sequence capture

**DOI:** 10.1093/bioadv/vbab033

**Published:** 2021-11-11

**Authors:** Sandeep K Kushwaha, Inger Åhman, Therése Bengtsson

**Affiliations:** 1 Department of Plant Breeding, Swedish University of Agricultural Sciences, Lomma 234 22, Sweden; 2 Bioinformatics, National Institute of Animal Biotechnology, Hyderabad 500 032, India

## Abstract

**Summary:**

The discovery of novel resistance genes (R-genes) is an important component in disease resistance breeding. Nevertheless, R-gene identification from wild species and close relatives of plants is not only a difficult but also a cumbersome process. In this study, ResCap, a support vector machine-based high-throughput R-gene prediction and probe generation pipeline has been developed to generate probes from genomic datasets. ResCap contains two integral modules. The first module identifies the R-genes and R-gene like sequences under four categories containing different domains such as TIR-NBS-LRR (TNL), CC-NBS-LRR (CNL), Receptor-like kinase (RLK) and Receptor-like proteins (RLPs). The second module generates probes from extracted nucleotide sequences of resistance genes to conduct sequence capture (SeqCap) experiments. For the validation of ResCap pipeline, ResCap generated probes were synthesized and a sequence capture experiment was performed to capture expressed resistance genes among six spring barley genotypes. The developed ResCap pipeline in combination with the performed sequence capture experiment has shown to increase precision of R-gene identification while simultaneously allowing rapid gene validation including non-sequenced plants.

**Availability and implementation:**

The ResCap pipeline is available at http://rescap.ltj.slu.se/ResCap/

**Contact:**

sandeep.kushwaha@slu.se or sandeep@niab.org.in

**Supplementary information:**

Supplementary materials are available at *Bioinformatics Advances* online.

## 1 Introduction

Plant breeding efforts to develop resistant varieties do still mainly rely on the introgression of major dominant disease or pest resistance genes (R-genes) from resistant cultivars or from landraces through repeated backcrossing. R-genes play a key role in the recognition of specific pathogen effector molecules, leading to an induction of plant defence signalling often associated with local hypersensitive response at the infection site (McHale *et al.*, 2006). Based on current knowledge, plant R-genes can be divided into at least five major classes, such as coiled–coiled nucleotide-binding leucine-rich repeat (CNL), Toll/interleukin-1 receptor-nucleotide-binding site leucine-rich repeat (TNL), Receptor-like kinase (RLK) and Receptor-like protein (RLP), and others (Sanseverino *et al.*, 2013). One strategy to improve the efficiency and durability of resistance is to stack R-genes and precede the rapidly evolving effector genes in pathogens. However, finding of R-genes from landraces and close relatives to crops is a difficult and laborious process. In this context, the SeqCap technique can make it possible to target regions of interest, while minimizing the fraction of off-targets at a large scale. The SeqCap technique picks up nucleotide fragments of interest from genomic and transcriptomic pools through a user-designed set of probes. Recently, the sequence capture technique has been used successfully for R-gene enrichment sequencing (RenSeq) in potato (Witek *et al.*, 2016), tomato (Andolfo *et al.*, 2014; de Oliveira *et al.*, 2018) and wheat (Steuernagel *et al.*, 2016; Zhang *et al.*, 2020). 

Mostly, sequence and motif similarity, domain matching and domain association-based methods are in use for resistance gene identification such as Disease Resistance Analysis and Gene Orthology (DRAGO) pipeline (Sanseverino *et al.*, 2013), R-gene analogues pipeline (RGAugury) (Li *et al.*, 2016) and NLR-parser (Steuernagel *et al.*, 2015). Prediction of R-proteins on the basis of sequence and domain similarity with a small set of reference R-genes is challenging due to the high level of diversity, as R-genes are under high selection pressure to adapt their immunity to the rapidly evolving effector genes in the pathogens (Marone *et al.*, 2013). R-gene identification from a plant species or landraces through traditional methods would be difficult to perform at large scale. But presently, a large number of plant genomes and transcriptomes have been sequenced and assembled. Despite the availability of draft genome and genome sequences, R-gene identification and validation are still difficult due to poor gene annotation model. However, machine learning techniques-based webservers and tools such as NBSPred (Kushwaha *et al.*, 2016) and DRPPP (Pal *et al.*, 2016) enabled *insilico* exploration of R-genes. However, the prediction results of these tools were never validated experimentally. Here, as an integrated solution, ResCap an automated pipeline has been developed for R-gene identification, nucleotide sequence extraction of R-gene from genome and transcriptome sequences, and probe generation to perform experimental validation.

## 2 Methods

**Fig. 1.**
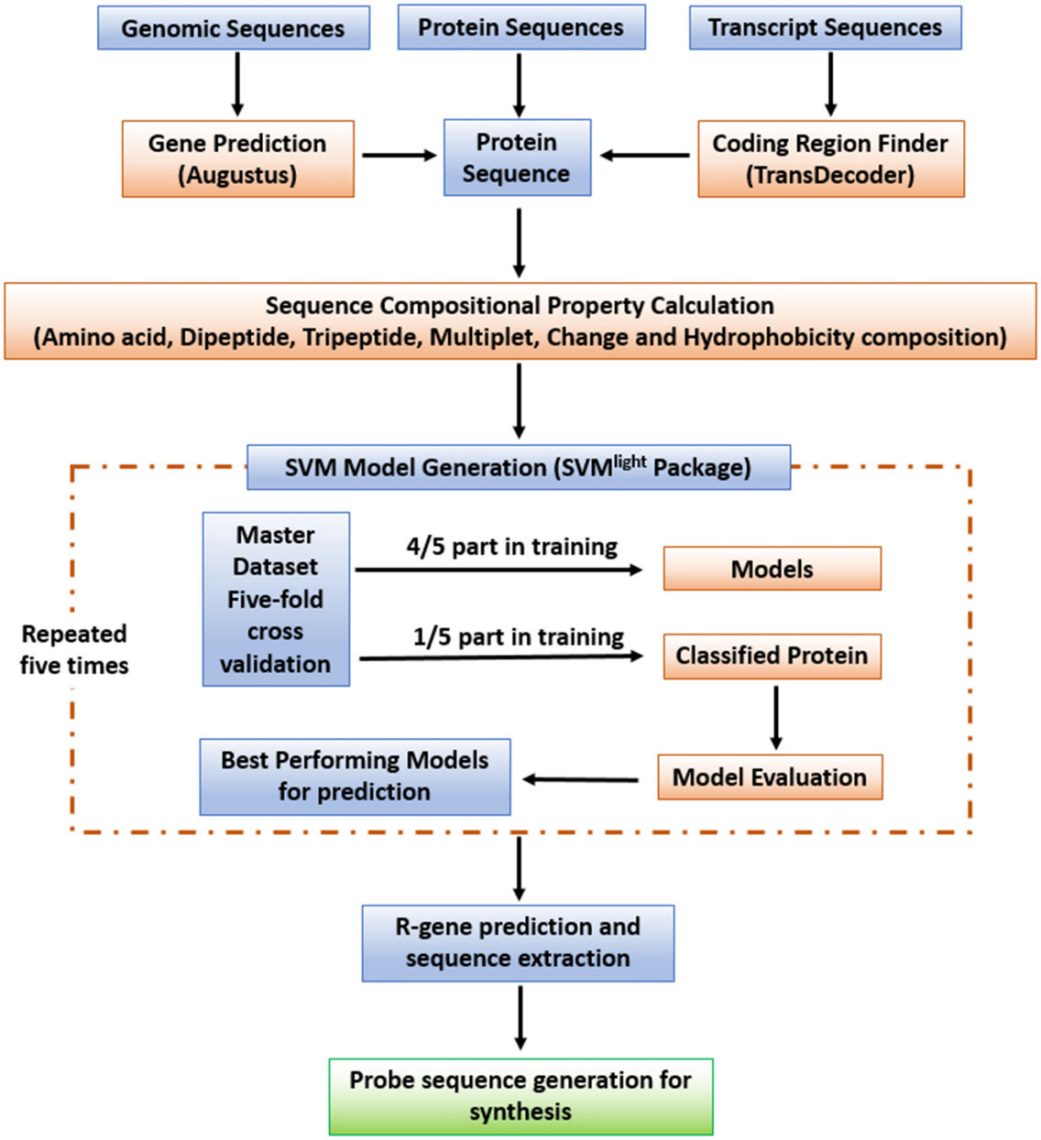
ResCap data processing workflow for R-gene identification and probe generation

R-gene and non-R-gene sequences were retrieved from public databases such as NCBI, Uniprot and PRGdb. Redundancy removal among extracted sequences was performed through clustering. A domain-based approach was used to generate the final datasets referred to as the positive and negative dataset. R-gene classes were identified among extracted sequences on basis of the occurrence of well-known R-gene domains such as NB-ARC, TIR, CC, kinase, LRR, Serine/threonine-LRR and Kinase-LRR. Sequences containing these domains are referred to as the positive dataset, whereas the negative dataset included all kind of sequences except R-gene and R-gene like sequences. Sequence compositional frequencies (amino acid frequency, dipeptide frequency, tripeptide frequency, multiplet frequency, charge and hydrophobicity composition) were calculated (Supplementary File Section S2), and all the calculated properties were gathered as a numerical feature vector for each sequence of the positive and negative dataset (Chaudhuri *et al.*, 2011; Ramana and Gupta, 2010). The SVM^light^ package modules (SVM_learn and SVM_classify) (Joachims, 1999) were used to generate SVM classifier for R-gene prediction. Best binary classifiers for each family were identified through 5-fold cross-validation technique (Supplementary File Section S3). Augustus gene prediction software was used in the pipeline for the annotation of plant genome (Stanke and Morgenstern, 2005). TransDecoder (Grabherr *et al.*, 2011) was used to generate protein sequences from transcripts. The flowchart of the pipeline is given in Figure  1. For the validation of ResCap pipeline, coding sequences of plants of poaceae family from the Gramene database (Gupta *et al.*, 2016) were extracted and processed through the ResCap pipeline and generated probes were synthesized using SeqCap EZ HyperCap, Nimblegen, Roche, USA. Six spring barley genotypes (142-31, 142-93, 252-33, 252-61, Barke and Lina) were selected for the experimental validation (Åhman and Bengtsson, 2019).

All the genotypes were grown under highly controlled experimental conditions (Supplementary Section S5) and homogenized leaf samples were used for RNA extraction. Library preparation, sequence capture experiment and sequencing were performed at Centre for Genomic Research, University of Liverpool, UK, and bioinformatics analysis was performed at Swedish University of Agricultural Sciences, Sweden. Generated sequence data are available at NCBI SRA public repository (PRJNA740109).

## 3 Implementation

Dell PowerEdge T440 Server E5-2430 with 16 core processors of 2.1 GHz, running on Ubuntu 20.04 LTS was used to host ResCap pipeline, and freely accessible as a web interface which was developed in PHP version 8.0. ResCap pipeline provides email confirmation for each submission and email notification upon job completion.

## 4 Results and discussion

A total of 1694 (CNL: 447; TNL: 515; RLK: 355; RLP: 377) sequences were involved in the training of four classes of R-gene family. Composition-based amino acid frequencies were used for numerical encoding of training sequences (Supplementary Section S2). In order to find best classifier for each R-gene class, 1176 binary models were created through sequential input of different kernel function and kernel associated parameters for model generation. Polynomial kernel associated d and C parameters were increased stepwise through a combination of 1, 2, 3, 4 … to … 9 for the d, and 10^−7^, 10^−6^ … to … 10^13^ for C whereas radial basis function kernel parameter gamma (g) was incremented stepwise 10^−15^ … to … 10^3^, and parameter C from 10^−5^ … to … 10^15^ (Kushwaha *et al.*, 2016). The mean Matthews correlation coefficient and prediction accuracy of the best-performed model, kernel type and kernel associated values are provided in Supplementary File (Table S3). ResCap prediction accuracy was compared with NLR-parser (Supplementary Tables S3–S6) and ResCap has detected higher number of sequences with R-protein domains than NLR-parser. Sequence capture experiment was performed to validate ResCap generated probes. Sequence capture data of six genotypes (142-31, 142-93, 252-33, 252-61, Barke and Lina) were evaluated, and bioinformatics analysis of sequence captured data is given in Supplementary File (Tables S8 and S9). On average, approximately 5 million high-quality paired-end reads were captured for each genotype by using designed probes. Both the pairs were merged and used for BLASTn similarity search against nucleotide sequences used for probe design. Among all captured reads, 27%, 71%, 4% and 0% reads were belonging to the CNL, RLK, RLP and TNL class, respectively. R-gene classes were analysed against the barley genome to identify common and uniquely expressed R-genes among barley genotypes (Supplementary File Figure S2). ResCap pipeline will be highly useful to develop a holistic understanding of disease susceptibility and resistance in crop varieties against pests and pathogens.

## Supplementary Material

vbab033_Supplementary_Data
