# Supplementary material for: ResCap: plant resistance gene prediction and probe generation pipeline for resistance gene sequence capture
Source: Bioinform Adv. 2021 Nov 11;1(1):vbab033. doi: 10.1093/bioadv/vbab033 (PMC9710708; doi:10.1093/bioadv/vbab033)
Supplement: vbab033_Supplementary_Data [file vbab033_supplementary_data.docx]

**Supplementary information for research article entitled “ResCap: Plant resistance gene prediction and probe generation pipeline for resistance gene sequence capture”**

**1. Data collection and pre-processing for resistance gene (R-gene) prediction model generation**

To create a positive dataset (collection of R-gene sequences), manually curated reference resistance nucleotide and protein sequences from the PRGdb database (Sanseverino, *et al*., 2013) were used to extract similar protein and nucleotide sequences from the NCBI public database through BLAST and Hidden Markov Model (HMM) profile similarity search using the hmmer package (Eddy, 2011). A local BLAST database was set up to perform similarity search, whereas a HMM profile (Eddy, 2011) was generated from reference sequences to explore R-gene sequences in the public domain.

**Table S1:** Number of manually curated reference R-gene nucleotide and protein sequences from PRGdb.

| **R-gene Class** | **Protein** | **Nucleotide** |
| --- | --- | --- |
| CC-domain-containing nucleotide-binding site leucine-rich repeat (CNL) | 50 | 50 |
| Kinase | 1 | 1 |
| Nucleotide-binding site leucine-rich repeat (NL) | 4 | 4 |
| Other | 19 | 19 |
| Receptor like kinase ( RLK) | 10 | 10 |
| Receptor like protein ( RLP ) | 12 | 12 |
| TIR-domain-containing nucleotide-binding protein (TN) | 1 | 1 |
| Toll/interleukin-1 receptor-domain-containing nucleotide-binding site leucine-rich repeat (TNL) | 15 | 15 |

Sequence ids for reference R-gene like similar sequences were extracted with the 90% sequence query coverage and 1e^-10^ e-value cut-off for the BLAST, whereas e-value cut-off 1e^-10^ was used for HMM profile search. These sequences were extracted to create the positive dataset. Additionally, a negative dataset (sequences without R-gene domains) was generated from public domain sequences and contained 313,862 plant protein sequences other than resistance proteins such as ribosomal proteins, metabolic proteins and other intracellular proteins etc. To create the final training dataset, a domain based approach was used for selection of sequences from both the positive and negative dataset. A pfam domain (Finn, *et al*., 2008) search was performed for both datasets and the criteria of presence or absence of R-protein domains were adopted for the sequence selection for both datasets. In the positive dataset, sequences contained various domains and motifs such as disease resistance protein signature, NB-ARC domain, TIR domain, leucine rich repeats, leucine rich repeat N-terminal domain, leucine-rich repeat signature, AAA and ABC ATPase, EGF_CA, Fer4_NifH, protein kinase domain, RX-CC_like, STKc_IRAK, tetratricopeptide repeat and wall-associated receptor kinase galacturonan-binding. All sequences containing any of the positive dataset domains were removed from the negative dataset and the resulting 43,285 sequences were used for SVM model generation. All extracted R-gene sequences were made non-redundant through the CD-HIT software (Li and Godzik, 2006). All sequences in the final dataset were 50% diverse to introduce more diversity among sequences for training. The number of R-gene sequences are provided in table S2.

**Table S2:** Number of extracted nucleotide and protein sequences for major R-gene classes

| **Resistance gene Class** | **Nucleotide** | **Protein** |
| --- | --- | --- |
| CNL | 447 | 447 |
| TNL | 515 | 515 |
| RLP | 355 | 355 |
| RLK | 377 | 377 |

**2. Calculation of compositional properties as numerical features for SVM training**

Six compositional attributes were calculated for the positive and negative dataset sequences. Attributes and their description are as follow (Chaudhuri, *et al*., 2011) (Ramana and Gupta, 2010):

1. **Amino acid Frequency (AAF)**

fi(a) = X_i_/L . . . . . . (1)

Where, X_i_ is the counts of i^th^ amino acid (i =1……... 20),

and L is the length of the protein sequence.

1. **Dipeptide Frequencies (DPF):** The frequency of a dipeptide (i,j)

DPF(f_ij_) = [(counts of ij^th^ dipeptide )∗ 100/ (L − 1) ]/2 . . . . . . (2)

Where, i,j =1, ..., 20 for each of 20 amino acids,

and L is the length of the protein sequence.

1. **Tripeptide Frequencies (TPF):** The frequency of a tripeptide (i,j,k)

TPF(fijk) = [(counts of ij^kth^ tripeptide) ∗ 100/ (L − 2) ]/3 . . . . . . (3)

Where, i, j, k =1, ..., 20 for each of 20 amino acids

and L is the length of the protein sequence.

1. **Multiplet Frequencies (MPF):**

Multiplets are homo-polymeric stretches of (X_n_), where X is the amino acid and n (integer) ≥ 2. After identifying all multiplets, the frequencies of the amino acids in the multiplets were computed as follow:

MPF fi(m) =Xmi/L . . . . . . (4)

Where, Xmi is the count of i^th^ amino acid occurring as multiplet, and L is the length of the sequence. There are 20 possible values for f_i_(m) for 20 amino acids.

1. **Hydrophobicity Composition (HC):**

Hydrophobic frequencies of amino acids (A, M, C, F, L, V, I) were calculated by,

$$f_{n=\frac{(counts of any of the seven hydrophobic amino acids)}{L}}$$

…… (5)

Where, L is the length of the sequence.

Moments (Mr) of the positions of hydrophobic amino acids were calculated for the characteristics of the distribution of these amino acids in a given protein sequence.

Mr = r^th^ order moment of the positions of hydrophobic amino acids

$$=\sum\frac{(X_{i}-X_{m})^{r}}{N}, \ldots\ldots.. (6)$$

Where X_m_ is the mean of all positions of charged amino acids,

$$X_{m}=\sum_{i=1}^{N} X_{i}/N$$

*X_m_* is the mean of sequence positions of all hydrophobic amino acids, *Xi* is the sequence position of the i^th^ hydrophobic amino acid where i is any of the 7 hydrophobic amino acids A, M, C, F, L, V, I; and N is the total number of hydrophobic amino acids in the sequence and r is from 2-5. The values of the r^th^ order moments were downscaled to smaller decimal values by dividing by (1000)^r^ while preparing the feature input to SVM.

1. **Charge Composition (CC):**

It is the fraction of charged amino acids divided by the length of the protein. Moments (Mr) of the positions of charged amino acids (R, K, D and E) from order 2–19 are calculated using the expression:

*Mr*$=\sum\frac{(X_{i}-X_{m})^{r}}{N}$ …………….. (7)

Where, Xm is the mean of all positions of charged amino acids, Xm =$\sum_{i}^{N} =1$  X_i_/N; X_i_ is the

position of i^th^ charged amino acid while N is the total number of charged amino acids in the sequence. The fractions of positively and negatively charged amino acids together with 18 moments yield a fixed length input vector of 20 dimensions.

**3. SVM model construction using the SVM^light^ package**

Support vector machine is a well-known supervised learning algorithm which have been used to identify the correct label for unknown datasets i.e. classification. SVM machine learning technique generates an optimal hyperplane which can divide the positively and negatively labeled datasets with the maximum margin of extrication to generate classification model. SVM utilizes an iterative training algorithm to create an optimal hyperplane, which is used to minimize an error function. The SVM technique has a number of optimization parameters for kernel functions such as linear, sigmoid, polynomial, and radial basis function (RBF) to facilitate optimization for classification during training. In this study, the SVM technique was implemented using software package SVM^light^ to generate classification models (Joachims, 1999).

**3.1 Performance evaluation**

To assess the performance of the prediction models, three threshold dependent parameters; specificity (SP), sensitivity (SN) and Matthews correlation coefficient (MCC) were calculated to identify the prediction performance of generated models (Ansari *et al*., 2008; Bhasin *et al*., 2004).

$$Sensitivity= \frac{TP}{TP+FN} \times100$$

$$Specificity= \frac{TN}{TN+FP} \times100$$

$$Accuracy= \frac{TP+TN}{TP+FP+TN+FN} \times100$$

$$MCC= \frac{\left( TP\times TN \right)-(FN\times FP)}{\sqrt{(TP+FN)}\times\left( TN+FP \right)\times\left( TP+FP \right)\times(TN+FN)} \times100$$

Where, TP: True positive; TN: True negative;

FP: False Positive; FN: False negative;

MCC value equal to 1 is regarded as a perfect prediction, whereas 0 is for a completely random prediction.

**3.2 Generated results**

Six compositional properties were used to generate the classification model and all six properties were calculated for each sequence of the positive and negative dataset. All properties were converted into a compositional properties vector for each sequence and a master dataset (combined compositional vector of positive and negative dataset) was generated. Total, 1176 models were generated through different combinations of kernel function and their parameters. The values for d and C were incremented stepwise through a combination of one to nine for d, and 10^-7^...to...10^13^ for C. For the radial basis kernel function, the gamma (g) parameter was incremented stepwise 10^-15^ to 10^3^ and parameter C from 10^-5^ to 10^15^. SVM prediction accuracy was well-known for binary classification. Therefore, four individual binary classifiers was generated for the identification of four classes of R-genes and five-fold cross validation was used to assess the performance of the method. Before training, dataset was randomized, and the positive and negative dataset were divided into five parts. Each part were containing nearly equal number of sequences for training and testing for each class. In each time, four different subsets were used for training and the remaining set for testing. Assuring that testing was performed on those proteins not included in the training set, a similar process was used for each class of protein separately. In the training dataset, positive and negative sample feature sets were labeled as +1 or -1 respectively. Positive and negative vectors were combined in alternate fashion for training, whereas test set sequences were labelled as 0. Total, 1176 models were generated from each set of the five training dataset. Good and poor performing models were identified during model performance evaluation through MCC value. Mean MCC values for each model were calculated across the five subsets and 169 models had a mean MCC greater than 0.95. The results from the ten best performing models are shown in Table S3. The final prediction output score for a given sequence was obtained by using all four class prediction models. Each sequence was processed for all the four classifier, but final class will be assigned with respect to highest SVM score.

**Table S3.** The best performing SVM models (classifier) to identify plant R-gene classes

| **R-gene class** | **Identified Models** | **Kernel Type** | **Mean MCC across the five test subset** | **Mean Accuracy (%)** |
| --- | --- | --- | --- | --- |
| CNL | 471 | RBF | 0.984 | 99.90 |
|  | 491 | RBF | 0.976 | 99.76 |
| RLK | 450 | RBF | 0.998 | 99.96 |
|  | 470 | RBF | 0.998 | 99.96 |
| RLP | 450 | RBF | 0.991 | 99.84 |
|  | 470 | RBF | 0.991 | 99.81 |
| TNL | 450 | RBF | 0.998 | 99.99 |

**3.3 ResCap R-gene prediction testing with an independent dataset**

To check the robustness of the R-gene prediction, an independent dataset was used. Sequences of the positive dataset which were not used in generation of classifier, were used to create an independent dataset. Independent data consisting of 5,541 NBS coding gene sequences only and is available in ResCap help section. ResCap prediction was compared with the NLRParser (Steuernagel, *et al*., 2015) for accuracy. NLR-Parser has given output for 5049 sequences (CNL: 2318, 437: TNL and 2294:N/A) from the independent dataset, but only 49.72% sequences have class assignment and domain description (Table-S4).

**Table-S4.** Comparison of ResCap and NLR-Parser prediction for independent dataset.

| **Dataset** | **NLR-Parser** | **ResCap Prediction** |
| --- | --- | --- |
| Independent Dataset (5541) | 2318(CNL)  437 (TNL) | 2786(CNL)  1462(TNL) |
| Accuracy (%) | 49.72%(Class assignment and domain description) | 76.64 %( Class assignment and domain description) |

Total, 2294 sequences were assigned as N/A by NLR-Parser for the independent dataset. Among all N/A assigned sequences, none of the sequences have NB-ARC annotation or the NBLRR signal annotations despite the presence of LRR and linker motif (7, 8, 9, and 11). Motif number 4, 5, 6 and 10 were matched with majority of sequences (around 88%), three motifs (4, 5 and 10) were matched to 1135 sequences, four motifs (4, 5, 6 and 10) were matched to 718 sequences and three motifs (4, 5 and 6) were matched to 178 sequences. Presence of motif for these 88% sequences were not very consistent. Therefore, class N/A was assigned for sequences. In the evaluation of NLRParser for independent dataset, we found that the N/A assigned class are not significantly contributing to total accuracy and it can also be a possible source of false positive prediction (Table – S4). Web-link of NBSPred and DRPPP tools were not found active at time of comparison of prediction results. ResCap and NBSPred both are SVM based high throughput prediction pipelines which are developed by first author. NBSPred was developed to identify only NBS-LRR coding genes. ResCap is an extension of the NBSPred pipeline for the identification of four classes of proteins (CNL, TNL, RLP, and RLK).

**3.4 ResCap testing with a PRGdb dataset**

PRGdb (http://prgdb.crg.eu/wiki/Main_Page) is the most comprehensive, web accessible, R-gene database for plants. We downloaded three different sequence files for R-gene (reference R-Genes, putative R-Genes, and contributed R-Genes) and R-protein (reference R-proteins, putative R-proteins, and contributed R-proteins) from PRGdb for the comparison of prediction accuracy. Each downloaded files contains sequences from all classes of R-gene. These sequence categories are made and maintained by PRGdb. Sequence count difference was observed in putative gene and protein category. However, number of protein and gene sequences in PRGdb annotation for deposited sequences are same. However, prediction comparison was made only for annotated PRGdb sequences. A comparison of ResCap, NLR-Parser prediction and PRGdb annotation is provided in Table-S5. All the numbers given for ResCap prediction is based on the counting of R-protein domains.

**Table-S5.** Comparison of ResCap prediction with PRGdb dataset.

| **Sequence Type** | **PRGdb sequences** | **PRGdb Annotation for deposited sequences** | **NLR-Parser Prediction**  **(CNL+ TNL)** | **ResCap**  **Prediction**  **(CNL+ TNL)** | **ResCap**  **Prediction (CNL+TNL**  **+RLK+RLP)** |
| --- | --- | --- | --- | --- | --- |
| All reference genes | 112 | 112 | 70 | 68 | 88 |
| All reference proteins | 112 | 112 | 70 | 68 | 88 |
| All putative genes | 103,067 | 41,523 | 16,590 | 16,355 | 37,453 |
| All putative proteins | 102,318 | 41,523 | 16,590 | 16,580 | 38,358 |
| All contributed genes | 2,474 | 2,474 | 280 | 322 | 900 |
| All contributed proteins | 2,474 | 2,474 | 584 | 650 | 1720 |

In ResCap, sequence composition-based properties were used for model generation for the prediction of different R-gene classes. Any sequence having high compositional similarity, but without R-gene domain, will be identified as R-gene in a particular class. A careful manual inspection is required for these kind of sequences to assign them into an R-gene class.

**4. R-gene nucleotide sequence extraction and probe generation**

After the identification of R-genes, nucleotide sequences of predicted genes will be extracted from the submitted genome or transcriptome sequences. Extracted nucleotide sequences will be used to generate probe sequences for sequence capture experiments. In ResCap pipeline, a well-established MetCap approach was used for probe generation in the performed sequence capture experiment. MetCap uses a hybrid approach for large-scale data set processing and probe design with any set of user-defined sequences. The probes generated by using the MetCap approach are not affected by differences in abundance to sequences due to clustering-based probe generation and selection approach to reduce the number of probes. MetCap probe selection approach is focused to maximize the coverage of individual cluster to enhance the hybridization chances. All generated clusters will produce the same number of probes with similar properties as defined by the user. The optimal number of probes is also an important factor for targeted sequence capture, but it is very hard to speculate on the exact number of probes for the capturing of a whole cluster, especially large sequence length cluster (Kushwaha, *et al*., 2015). ResCap can generate probes for each cluster from different regions to facilitate efficient capturing. After processing, ResCap produces a number of output files as presented in Table S7.

**Table S7** List of ResCap generated files and their descriptions

| **S.N.** | **File Name** | **File Description** |
| --- | --- | --- |
| 1. | RPred Results | R-gene prediction results |
| 2. | Filename.CDS_Ext_RgeneSeq.fn  Filename.PEP_Ext_RGeneSeq.fn | Protein and nucleotide sequences of predicted R-genes |
| 3. | ProbeList_For_Synthesis.txt | Probe sequences for synthesis |
| 4. | Filename.probe_synthesis_cycle.txt | Count of probe synthesis cycle for generated probes |
| 5. | Filename.probe_run_summary. | Run summary for all predicted R-gene sequences |

**5. Plant material, growth conditions, RNA extraction and bioinformatics analysis**

Six spring barley genotypes (142-31, 142-93, 252-33, 252-61, Barke and Lina) were selected for the validation of generated resistance gene probes (Åhman and Bengtsson, 2019). Seeds of all the genotypes were first germinated on moist filter paper in a refrigerator (4–8°C) for three days, followed by two days at room temperature. Later, seedlings were planted in plastic pots with a diameter of 10 cm using Emmaljunga exklusiv Blom & Plantjord soil (Emmaljunga torvmull AB, Vittsjö, Sweden). All genotypes were grown in a climate controlled growth chamber at 20°C, with a photoperiod of 16 h light and 8 h dark and a relative humidity of 80%. Metal halogen lamps resulted in a PAR of 200μmol m−2s−1 at plant level at the Swedish University of Agricultural Sciences, Sweden. Aphid infestation was performed as described in Kim *et al*. (2020). At 15 days after planting 20 aphids (*Rhopalosiphum padi* L.) were added into a cage placed at the midsection of the second leaf of each plant. Controls consisted of plants with an empty cage. Leaf samples were taken at 6 and 30 hour post aphid infestation (hpi) from three replicates (plants) per genotype and per treatment (+/- aphids). At sampling, aphids were carefully removed from the leaf with a soft brush. Thereafter, the leaf was immediately frozen in liquid nitrogen and stored at −80˚C until RNA extraction.

RNA from the homogenized leaf samples were extracted using the RNeasy Mini Kit (Qiagen GmbH, Hilden, Germany) as previously described in Kim *et al*. (2020). The integrity and quality was checked using a ND-1000 NanoDrop (Wilmington, USA) and gel electrophoresis (1.2% E-Gels® Life Technologies, Carlsbad, CA). RNA integrity number (RIN) value of samples were between 6 and 8 for cDNA synthesis. Library preparation, sequence capturing and sequencing were performed at Centre for Genomic Research, University of Liverpool, UK. Paired end sequencing was performed through Illumina novoseq platform. Forward and reverse end reads were merged through pear software (Zhang, *et al*., 2014) and resulted sequences were used for bioinformatics analysis at the Swedish University of Agricultural Sciences, Sweden. The bioinformatics analysis results of the sequence captured data for the selected genotypes at 6hr without aphid infestation are given in Table S8 and S9, and number of unique and commonly identified genes with respect to genotypes are given in Figure S1.

# Table S8. Quality control results of captured sequences of the barley genotypes. Presented values are mean values of three plant replicates.

| **Genotype** | **142-31** | **142-93** | **252-33** | **252-61** | **Barke** | **Lina** |
| --- | --- | --- | --- | --- | --- | --- |
| High quality clean reads | 4,911,450 | 5,238,746 | 3,586,417 | 4,707,824 | 5,172,146 | 5,105,094 |
| Merged reads | 3,555,637 | 3,846,022 | 2,620,526 | 3,414,785 | 3,763,486 | 3,672,339 |
| Base-pair count (bps) | 693,341,204 | 739,509,128 | 506,420,295 | 661,987,158 | 727,196,207 | 716,965,633 |
| Mean sequence length (bps) | 194.998 | 192.279 | 193.251 | 193.859 | 193.224 | 195.234 |

**Table S9.** Comparative table of BLASTn hits and capture efficiency against local used databases for probe generation using e-value cut off of 1e^-10^

| **S.N.** | **Descriptions** | **142-31** | **142-93** | **252-33** | **252-61** | **Barke** | **Lina** |
| --- | --- | --- | --- | --- | --- | --- | --- |
| 1. | Assembled Reads | 3,555,637 | 3,846,022 | 2,620,526 | 3,414,785 | 3,763,486 | 3,672,339 |
| 2. | Targeted Nucleotide Database (BLASTN) | 3,258,065 | 3,553,517 | 2,391,853 | 3,118,807 | 3,410,683 | 3,396,220 |
| 3. | Reads without mismatch  (100% query coverage) | 616,165 | 655,753 | 420,712 | 546,964 | 602,331 | 609,042 |
| 4. | Captured reads counts for gene families (%) | | | | | | |
|  | CNL | 168,394  (27%) | 182,939  (27%) | 93,063  (22%) | 119,098  (21%) | 140,131  (23%) | 176,314  (28%) |
|  | TNL | 370 (0%) | 372 (0%) | 307 (0%) | 378 (0%) | 468 (0%) | 334 (0%) |
|  | RLK | 431042  (69%) | 453759  (69%) | 309629  (73%) | 403253  (73%) | 435664  (72%) | 417712  (68%) |
|  | RLP | 16359  (2%) | 18683  (2%) | 17713  (4%) | 24235  (4%) | 26068  (4%) | 14682  (2%) |
| 5 | Identified number of unique sequences for gene families | | | | | | |
|  | CNL | 185 | 189 | 173 | 163 | 172 | 196 |
|  | TNL | 4 | 3 | 3 | 5 | 4 | 3 |
|  | RLK | 348 | 343 | 335 | 337 | 343 | 337 |
|  | RLP | 22 | 27 | 28 | 28 | 28 | 27 |


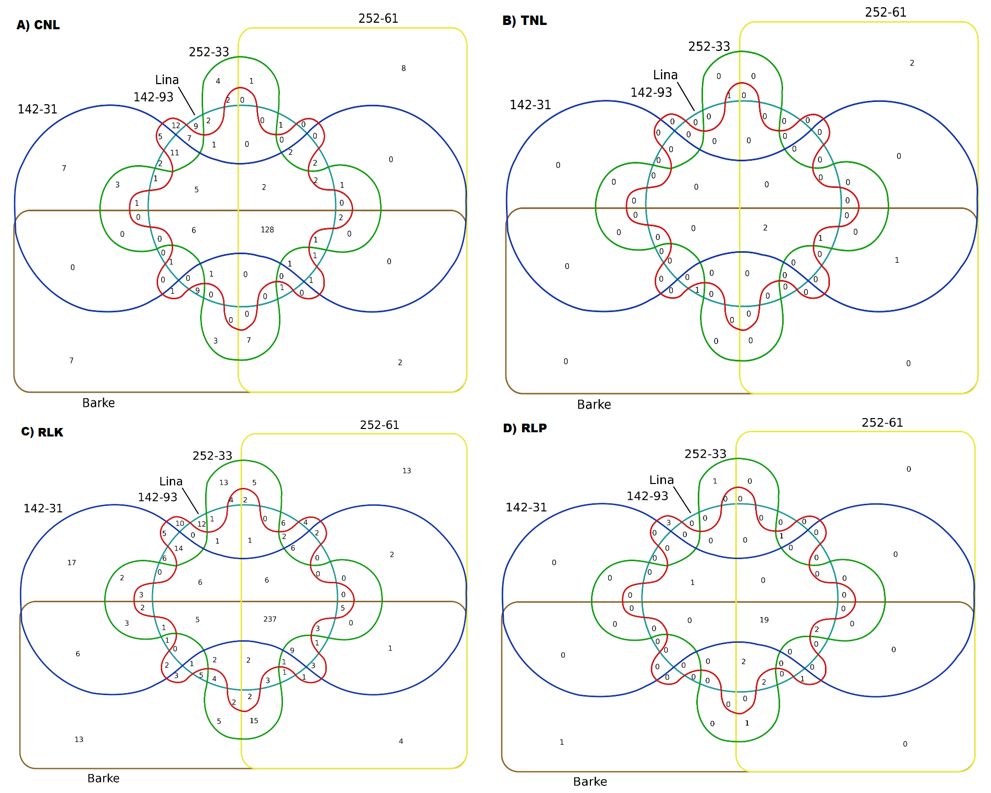


**Figure S1:** Venn diagrams of identified R-genes (CNL, TNL, RLK and RLP) from sequence capture data in six barley genotypes (142-31, 142-93, 252-33, 252-61, Barke and Lina).

**References**

Åhman, I. and Bengtsson, T. Introgression of resistance to Rhopalosiphum padi L. from wild barley into cultivated barley facilitated by doubled haploid and molecular marker techniques. *TAG. Theoretical and applied genetics. Theoretische und angewandte Genetik* 2019;132(5):1397-1408.

Chaudhuri, R.*, et al.* FungalRV: adhesin prediction and immunoinformatics portal for human fungal pathogens. *BMC Genomics* 2011;12(1):192.

Eddy, S.R. Accelerated Profile HMM Searches. *PLOS Computational Biology* 2011;7(10):e1002195.

Finn, R.D.*, et al.* The Pfam protein families database. *Nucleic acids research* 2008;36(Database issue):D281-288.

Kushwaha, S.K.*, et al.* MetCap: a bioinformatics probe design pipeline for large-scale targeted metagenomics. *BMC Bioinformatics* 2015;16(1):65.

Kim S.Y. *et al*. Mutations in Two Aphid-Regulated β-1,3-Glucanase Genes by CRISPR/Cas9 Do Not Increase Barley Resistance to Rhopalosiphum padi L. Frontiers in Plant Science 2020; 11(1043).

Li, W. and Godzik, A. Cd-hit: a fast program for clustering and comparing large sets of protein or nucleotide sequences. *Bioinformatics (Oxford, England)* 2006;22(13):1658-1659.

Ramana, J. and Gupta, D. FaaPred: a SVM-based prediction method for fungal adhesins and adhesin-like proteins. *PLoS One* 2010;5(3):e9695-e9695.

Sanseverino, W.*, et al.* PRGdb 2.0: towards a community-based database model for the analysis of R-genes in plants. *Nucleic acids research* 2013;41(Database issue):D1167-1171.

Steuernagel, B.*, et al.* NLR-parser: rapid annotation of plant NLR complements. *Bioinformatics (Oxford, England)* 2015;31(10):1665-1667.

Zhang, J.*, et al.* PEAR: a fast and accurate Illumina Paired-End reAd mergeR. *Bioinformatics (Oxford, England)* 2014;30(5):614-620.
